# 

**Published:** 1888-09

**Authors:** 


### BOOK NOTICES.

THE CENTURY MAGAZINE is a standard monthly, which, once becoming used to, we cannot do without. The history of Lincoln and his times, bids fair to be the standard account of the troubleous days of the great rebellion and the great men who saved the union from dismemberment. Its Siberian papers increase in interest as they multiply, and give us many interesting details of that far off home of the Russian exile.

CORNELL UNIVERSITY.—The agricultural department of this well reputed institution gives us in its August bulletin, much valuable information in respect to the best foods for food-supplying animals, with the outcome skilfully illustrated in colors.

OUR DUMB ANIMALS.—The purpose of this worthy monthly is explained by its title. As a rule, we are apt to have too little consideration for those dumb helpers of the field and pets of the household, upon which our enjoyments so largely depend. "There is many a horse more deserving of immortality than the man who rides it ; there is many a dog that has more disinterested love than the man who owns it," said the late Henry Ward Beecher, and so say we. Published by the Massachusetts Society for the prevention of cruelty to animals, 19 Milk St., Boston, at 50 cents per annum.

Our thanks are due for the receipt at this office of the following papers :

A COMMENCEMENT, address, College of the City of New York. By President, J. Edward Simmons, L. L. D.

CHOLERA INFANTUM AND ITS TREATMENT, by H. F. Hendrix, M. D., St. Louis.

RECTAL INSUFFLATION OF HYDROGEN GAS, by Prof. N. Senn, M. D., P. H. D., of the Rush Medical College, Chicago.

VICK'S ILLUSTRATED MONTHLY has been so often praised by us for its excellent contents and richly colored flower pieces, that if it did not grow better and better every month, we should have long ago exhausted the subject. Nothing better of the kind. Send for specimen. James Vick, Seedsman, Rochester, N. Y.

THE INTERNATIONAL JOURNAL OF SURGERY AND ANTISEPTICS, a New York quarterly, 95 William St., New York City, $1 per annum, is a publication of unusual merit in its legitimate department.

